# Exendin-4 Preserves Blood-Brain Barrier Integrity *via* Glucagon-Like Peptide 1 Receptor/Activated Protein Kinase-Dependent Nuclear Factor-Kappa B/Matrix Metalloproteinase-9 Inhibition After Subarachnoid Hemorrhage in Rat

**DOI:** 10.3389/fnmol.2021.750726

**Published:** 2021-12-23

**Authors:** Zhiyi Xie, Budbazar Enkhjargal, Matei Nathanael, Lingyun Wu, Qiquan Zhu, Tongyu Zhang, Jiping Tang, John H. Zhang

**Affiliations:** ^1^Department of Neurosurgery, The Second Affiliated Hospital, Zhejiang University, Hangzhou, China; ^2^Department of Physiology and Pharmacology, Loma Linda University, Loma Linda, CA, United States

**Keywords:** AMPK, blood-brain barrier, Exendin-4, GLP-1r, subarachnoid hemorrhage

## Abstract

In this study, we investigated the role of Exendin-4 (Ex-4), a glucagon-like peptide 1 receptor (GLP-1R) agonist, in blood-brain barrier (BBB) disruption after subarachnoid hemorrhage (SAH) in rats. The endovascular perforation model of SAH was performed in Sprague-Dawley rats. Ex-4 was intraperitoneally injected 1 h after SAH induction. To elucidate the underlying molecular mechanism, small interfering ribonucleic acid (siRNA) for GLP-1R and Dorsomorphin, a specific inhibitor of adenosine monophosphate-activated protein kinase (AMPK), were intracerebroventricularly injected 48 h before induction of SAH correspondingly. Immunofluorescence results supported GLP-1R expressed on the endothelial cells of microvessels in the brain after SAH. Administration of Ex-4 significantly reduced brain water content and Evans blue extravasation in both hemispheres, which improved neurological scores at 24 h after SAH. In the mechanism study, Ex-4 treatment significantly increased the expression of GLP-1R, p-AMPK, IκB-α, Occludin, and Claudin-5, while the expression of p-nuclear factor-kappa B (NF-κB) p65, matrix metalloproteinase-9 (MMP-9), and albumin was significantly decreased. The effects of Ex-4 were reversed by the intervention of GLP-1R siRNA or Dorsomorphin, respectively. In conclusion, Ex-4 could preserve the BBB integrity through GLP-1R/AMPK-dependent NF-κB/MMP-9 inhibition after SAH, which should be further investigated as a potential therapeutic target in SAH.

## Introduction

Subarachnoid hemorrhage (SAH) is a feared disease with a high rate of mortality and disability (van Gijn et al., 2007; Nieuwkamp et al., 2009). Early brain injury (EBI) is considered a significant determinant of unfavorable outcomes following SAH (Sehba et al., 2012). Specifically, disruption of the blood-brain barrier (BBB) is a critical pathologic process of EBI and shows promise as a therapeutic target in ameliorating the detrimental effects of SAH (Chen et al., 2014).

Matrix metalloproteinase-9 (MMP-9), a member of zinc- and calcium-dependent proteolytic enzymes family,(Rosell and Lo, 2008) is widely distributed in multiple cell types such as neurons, astrocytes, microglia, and endothelial cells, across the central nervous system (CNS) (Kawasaki et al., 2008; Ying et al., 2016). MMP-9 has been shown to cause BBB disruption through the degradation of basal lamina proteins, tight junctions, and the extracellular matrix (Guo et al., 2010; Ying et al., 2016). The transcription of MMP-9 is endogenously regulated *via* its promoter binding site to nuclear factor-kappa B (NF-κB) (Bond et al., 2001). Thus, the regulation of NF-κB has therapeutic potential to downregulate MMP-9, prevent BBB disruption, and ameliorate the deleterious neurological damage of SAH.

Glucagon-like peptide 1 (GLP-1), a gut hormone released from intestinal L cells, is responsible for promoting insulin secretion after food intake in a glucose-dependent manner (Ahren, 1998; Parhofer, 2007). Exendin-4 (Ex-4) is a GLP-1 receptor (GLP-1R) agonist clinically approved to treat patients with adult type 2 diabetes (Briones and Bajaj, 2006). Several studies have supported the beneficial effects of GLP-1R agonists in central nervous system diseases, including both ischemic and hemorrhagic stroke (Teramoto et al., 2011; Hou et al., 2012; Rachmany et al., 2013; Kuroki et al., 2016; Zhu et al., 2016). A recent study of ischemic stroke in rats has reported the ability of Ex-4 to protect BBB integrity by inhibiting MMP-9 (Kuroki et al., 2016). Furthermore, in a hemorrhagic stroke model, Ex-4-mediated phosphorylation of adenosine monophosphate-activated protein kinase (AMPK) (Hou et al., 2012) is neuroprotective by suppressing downstream NF-κB signaling (Salminen et al., 2011).

Therefore, in this study, we hypothesize that Ex-4 is neuroprotective by reducing BBB damage of EBI through downregulation of downstream targets, NF-κB and MMP-9 *via* phosphorylation of AMPK (p-AMPK) after experimental SAH model in rats.

## Materials and Methods

### Animals

All experimental protocols in this study were approved by the Institutional Animal Care and Use Committee (nn) of Loma Linda University, were in accordance with the ARRIVE (Animal Research: Reporting *in vivo* Experiments) guidelines, and comply with the National Institutes of Health guidelines for the Use of Animals in Neuroscience Research. The adult male Sprague-Dawley rats (280–340 g, Harlan, IN, United States) used in this study were housed in a light and temperature-controlled room with unlimited access to food and water.

### Subarachnoid Hemorrhage Model

The endovascular perforation model has been described in detail in previous manuscripts (Duris et al., 2011). Briefly, rats were maintained in anesthesia with 3% isoflurane in 70/30% medical air/oxygen by a rodent ventilator (Harvard Apparatus, Holliston, MA, United States) after intubation. Devascularization of the left external carotid artery was performed to prepare a stump for further puncture. A sharpened 4–0 nylon suture was inserted into the left internal carotid artery through a small incision next to the stump. The suture was advanced until resistance was detected at the bifurcation of the anterior and middle cerebral artery. Then, an extra puncture was performed to perforate with an immediate withdrawal. The sham-operated group underwent the same procedure without endovascular puncture.

### Experimental Design

**FIGURE 1:**
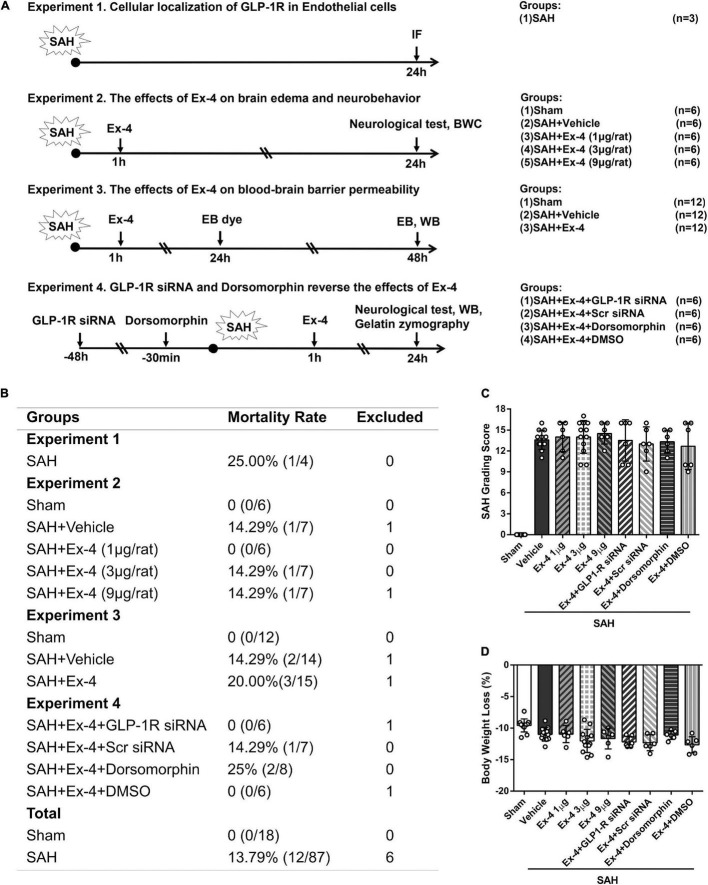
Basic characteristics of the study. **(A)** Experimental design and animal groups. **(B)** Animal usage and mortality rates of all groups at 24 h after SAH. **(C)** SAH grading scores and **(D)** body weight loss (%) of animals for brain water content (BWC) and Western blots. There is no significant difference in SAH grading scores and body weight loss among all SAH groups. GLP-1R, a glucagon-like peptide 1 receptor; SAH, subarachnoid hemorrhage; WB, western blot; IF, immunofluorescence; h, h; Ex-4, Exendin-4; Vehicle, 0.9% sterile NaCl; DMSO, dimethyl sulfoxide; Dorsomorphin, a specific inhibitor of AMPK; siRNA, small interfering ribonucleic acid; Scr siRNA, scrambled siRNA; BWC, brain water content; AMPK, adenosine monophosphate-activated protein kinase.

Animals were randomly assigned to the four separate experiments as described (Figure 1A).

### Experiment 1

To determine the endogenous GLP-1R expression in endothelial cells after SAH, 3 rats underwent SAH and double immunofluorescence staining of GLP-1R with von Willebrand factor (vWF), an endothelial cell marker, at 24 h after SAH.

### Experiment 2

To evaluate the effects of Ex-4 on brain edema and neurobehavioral function, 30 rats were assigned to five groups: sham, SAH + Vehicle (1 ml sterile 0.9% of NaCl), SAH + Ex-4 (1 μg) (1 μg in 1 ml sterile 0.9% of NaCl, Sigma-Aldrich, St. Louis, MO, United States), SAH + Ex-4 (3 μg) (3 μg in 1 ml sterile 0.9% of NaCl) (Lee et al., 2011; Kuroki et al., 2016), and SAH + Ex-4 (9 μg) (9 μg in 1 ml sterile 0.9% of NaCl) (*n* = 6 per group). Vehicle or Ex-4 was administered intraperitoneally (i.p.) at 1 h after SAH. SAH grading score, modified Garcia, and beam balance tests were assessed 24 h after SAH. According to these results, the 3 μg dose of Ex-4 was selected for the following experiments. Plasma glucose level was measured and recorded at 8-time points during the first 24 h after SAH.

### Experiment 3

To assess the effects of Ex-4 on BBB permeability, 36 rats were randomized into three groups: sham, SAH + Vehicle, and SAH + Ex-4 (*n* = 12 per group). Evans blue dye (2% in sterile 0.9% of NaCl; 4 ml/kg of body weight) was injected i.p. at 24 h after SAH and allowed to circulate for another 24 h before sacrifice (*n* = 6 per group) (Manaenko et al., 2011). Western blots were performed with the ipsilateral hemisphere samples at 24 h after SAH (*n* = 6 per group). SAH grading and neurological scores were evaluated 24 h after SAH in animals used for Western blots.

### Experiment 4

A total of 24 four rats were assigned randomly to 4 groups for the mechanism study: SAH + Ex-4 + GLP-1R small interfering ribonucleic acid (siRNA) (500 pmol of GLP-1R siRNA), SAH + Ex-4 + Scr siRNA (500 pmol of scrambled siRNA), SAH + Ex-4 + Dorsomorphin (10 mmol/L in 20% dimethyl sulfoxide [DMSO] of phosphate-buffered saline [PBS]) (An et al., 2015), and SAH + Ex-4 + DMSO (10 μl of 20% DMSO in PBS) (*n* = 6 per group). GLP-1R siRNA and scrambled siRNA were injected intracerebroventricularly (i.c.v) 48 h before SAH (Salahpour et al., 2007), while DMSO and Dorsomorphin were injected i.c.v. 30 min before SAH (An et al., 2015). Western blots (*n* = 6 per group) and gelatin zymography (*n* = 6 per group) were performed on the ipsilateral hemisphere samples of the brains collected in Experiments 3 and 4 at 24 h after SAH.

### Measurement of Blood Glucose Level

Plasma glucose levels of animals in Experiment 2 were measured before SAH, at 0 (immediately), 30, 60, 90, 120, 180 min, and 24 h after delivering the vehicle or drug (Kuroki et al., 2016). At each time point, the blood samples were taken from the tail vein and tested with a blood glucose meter (Ascensia Diabetes Care US Inc., NJ, United States).

### Subarachnoid Hemorrhage Grade

Photographs of the basal surface of the brain sample were captured immediately after euthanasia and evaluated blindly, following a previously described procedure (Sugawara et al., 2008). Briefly, the scores of six predetermined segments (scored from 0 to 3 in each segment) were assessed according to the amount of clotted subarachnoid blood. The total SAH grading score was calculated as the sum of six segments. A SAH grading score lower than 8 was considered as an indicator of exclusion for mild SAH (Sugawara et al., 2008).

### Neurological Evaluation

Modified Garcia and the beam balance tests were performed to assess neurological function 24 h after SAH, as previously described (Garcia et al., 1995; Suzuki et al., 2010). Six subtests of the modified Garcia test (spontaneous activity, spontaneous movement of four limbs, forelimb outstretching, vibrissa touch, body proprioception, and climbing capacity) were evaluated and recorded by an independent observer. To evaluate beam balance score, animals were put on a narrow wooden beam for a 1-min observation and scored according to their beam walking ability. The mean score from three consecutive trials (scored from 0 to 4) represented the final beam walking score of the targeted animal.

### Intracerebroventricular Administration

Intracerebroventricular drug administration was performed as previously described (Ramirez-Amaya et al., 2013). Animals were placed in a stereotaxic apparatus and maintained in anesthesia with 2.5% isoflurane in 70/30% medical air/oxygen. A burr hole (1.5 mm posterior and 0.9 mm lateral to bregma) was drilled on the right side of the skull following a scalp incision along the midline. The needle of a 10 μl Hamilton syringe (Microliter701; Hamilton Company, Reno, NV, United States) was inserted into the right lateral ventricle through the hole (3.3 mm below the horizontal plane of the bregma). Drugs and siRNA were delivered into the right lateral ventricle at a rate of 1 μl/min by a pump. The needle was kept in place for another 5 min before being retracted manually. Then, the burr hole was sealed with bone wax. GLP-1R siRNA (SR515617) and negative control scrambled RNA (SR30004) were prepared at 500 pmol in 5 μl of RNAse free suspension buffer (SR30005) (OriGene Technologies, Rockville, MD, United States) and infused (the siRNAs) 48 h before SAH induction in the SAH + Ex-4 + GLP-1R siRNA and SAH + Ex-4 + Scr siRNA groups, respectively. GLP-1R siRNA included a pool of four different siRNA duplexes to improve the knockdown efficiency. All siRNA sequences are provided in 5′→3′ orientation:

(a) GLP-1R siRNA-1 Sense: GGCAUUGUCAAGUAUCUC UACGAGG Antisense: CCUCGUAGAGAUACUUGACAAUGCC
(b) GLP-1R siRNA-2 Sense: GUUUCUGCCCUGCAUGG GUACUACT Antisense: AGUAGUACCCAUGCAGGGCAGAAAC
(c) GLP-1R siRNA-3 Sense: AUAUCUGUGGAUGGUGUU GAAGATA Antisense: UAUCUUCAACACCAUCCCAGAUAT
(d) Scrambled siRNA Sense: CGUUAAUCGCGUAUAAUA CGCGUAT Antisense: AUACGCGUAUUAUACGCGAU UAACGAC

Dorsomorphin dihydrochloride (Santa Cruz Biotechnology Inc., TX, United States), a selective AMPK inhibitor, also known as compound C, was prepared at 10 mmol/L in 20% DMSO of PBS and injected (10 μl) 30 min before SAH induction in SAH + Ex-4 + Dorsomorphin groups.

### Immunofluorescence Staining

The brain samples for immunofluorescence staining were prepared as previously described (Enkhjargal et al., 2014). Animals were intracardially perfused with 60 ml ice-cold PBS and 10% paraformaldehyde in deep anesthesia, and samples were collected at 24 h after SAH. After being fixed in 10% paraformaldehyde and 30% sucrose, the whole brain was stored in a freezer at −80°CC. Then, we cut the brains into 10 μm thick coronal sections using a cryostat (LM3050S; Leica Microsystems, Bannockburn, III, Germany). To perform double immunofluorescence staining, brain sections were incubated using the primary antibody of vWF (1:100, FITC, Abcam, Cambridge, MA, United States) and anti-GLP-1R (1:50, sc-390774, Santa Cruz Biotechnology Inc.) overnight at 4°C. After being incubated with anti-mouse secondary antibody (1:500, Jackson Immunoresearch, West Grove, PA, United States) at room temperature for 2 h, the sections were visualized and photographed with a fluorescence microscope (Leica Microsystems, Germany) for co-staining at × 400 magnification.

### Brain Water Content (Brain Edema)

Samples for brain water content (BWC) measurements were separated into four parts (left hemisphere, right hemisphere, cerebellum, and brain stem) and collected 24 h after SAH induction (Wu et al., 2016). Each part was weighed immediately after removal (wet weight) and kept in a 100°C oven for 72 h to measure dry weight. The percentage of BWC was calculated as [(wet weight–dry weight)/wet weight] × 100%.

### Evans Blue Extraction

A 2% solution of Evans blue in normal saline (4 ml/kg of body weight) was injected i.p. at 24 h after SAH. The stain was allowed to circulate for an additional 24 h before sacrifice. Then, animals were transcardially perfused with 120 ml of ice-cold PBS in deep anesthesia. The collected brain samples were divided into four parts, namely, right hemisphere, left hemisphere, cerebellum, and brain stem, and were stored in a freezer at −80°C. After homogenization of each sample with PBS, the solution was centrifuged for 30 min at 14,000 r/min in 4°C. The supernatant was collected, mixed with an equal amount of trichloroacetic acid, and incubated at room temperature for 1 h. Then, the sample was centrifuged for 30 min at 15,000 r/min in 4°C to separate the supernatant for measurements. Evans blue stain results were measured using a spectrophotometer (Thermo Spectronic Genesys 10 UV, Thermo Fischer Scientific Inc., Waltham, MA, United States) at 610 nm and quantified with a standard curve (Manaenko et al., 2011). The results are presented as a fold increase compared with the sham group.

### Western Blot Analysis

Western blot analyses were performed as previously reported (Sozen et al., 2009). The entire left hemisphere was isolated and collected for sample preparation 24 h after SAH (*n* = 6 per group). After a protein concentration adjustment, equal amounts of protein were loaded into each lane of an SDS-PAGE gel (7.5 to 12%) followed by electrophoresis. Next, samples were transferred onto a nitrocellulose membrane and blocked with a blocking solution for 2 h at room temperature. The membrane was incubated at 4°C overnight with a primary antibody: anti-IκB-α (1:1500), anti-NF-κB p-65 (1:1000), anti-MMP-9 (1:1000), anti-albumin (1:5000), anti-occludin (1:1500), anti-claudin-5 (1:1000) (Abcam, Cambridge, MA, United States), anti-p-AMPK (1:1000), anti-AMPK (1:1000), anti-p-NF-κB p-65 (1:1000) (Cell signaling, Boston, MA, United States), anti-GLP-1R (1:200), and anti-β-actin (1:5000) (Santa Cruz Biotechnology Inc.). Then, corresponding secondary antibodies (1:5000, Santa Cruz Biotechnology Inc., and anti-rabbit, EMD Millipore Corporation, MA, United States) were incubated with the membrane for 2 h at room temperature. Blot bands were visualized using an ECL reagent (Amersham Biosciences United Kingdom Ltd., PA, United States). Non-saturated bands were selected to perform densitometry quantification using Image J software (Image J 1.4, NIH, United States). Relative changes in protein expression were assessed by normalizing density to β-actin/total-AMPK/total-NF-κB p-65 accordingly.

### Gelatin Zymography for Measurement of Matrix Metalloproteinase Activity

Samples for gelatin zymography were prepared following Western blot procedures, but differed in sample preparation, using the zymogram sample buffer (Bio-Rad Laboratories, Hercules, CA, United States). About 40 μl of the sample was loaded per lane onto a polyacrylamide gel containing 0.1% gelatin (Sigma-Aldrich) for electrophoresis. Then, the gel was washed in renaturation buffer (Bio-Rad Laboratories) for 1 h. After rinsing with distilled water 10 times, the gel was incubated in development buffer (Bio-Rad Laboratories) at 37°C for 48 h. Staining was performed using Coomassie Brilliant Blue R-250 staining solution (Bio-Rad Laboratories) for 1 h and followed by a destaining solution (10% acetic acid and 40% methanol). The activity of the matrix metalloproteinases was demonstrated as clear zones in a blue background. Quantification analysis of the intensity was measured using Image J software (Image J 1.4) (Egashira et al., 2015). The activity of MMPs was represented as the intensity ratio of active form/proform.

### Statistical Analysis

Statistical analyses were performed using Graph Pad Prism (Graph Pad Software Inc., San Diego, CA, United States). All data were subjected to one-way analysis of variance (ANOVA) (two-way ANOVA was applied to analyze data of blood glucose level at different time points before/after SAH) followed by multiple comparisons between groups using Tukey’s *post hoc* test and represented as mean ± SD. A value of *P* less than 0.05 was considered statistically significant.

## Results

### Mortality, Subarachnoid Hemorrhage Grade, and Body Weight Loss

Of the 111 surgeries that we performed, 6 rats with mild SAH were excluded, according to previously published exclusion criteria (Sugawara et al., 2008). The overall mortality in all SAH groups was 13.79% (12/87, Figure 1B), while no animal died in the sham group. When we compared the SAH grading scores of all animals prepared for BWC measurement and western blots/gelatin zymography at 24 h after surgery, no significant difference was detected among the SAH groups (Figure 1C). Bodyweight loss (%) was calculated as the lost body weight/body weight before surgery × 100% in same animals, and administration of Ex-4 (1, 3, and 9 μl per rat), siRNAs, and Dorsomorphin did not significantly increase body weight loss compared with SAH + Vehicle group (Figure 1D).

### Expression of Glucagon-Like Peptide 1 Receptor in Endothelial Cells After Subarachnoid Hemorrhage

**FIGURE 2:**
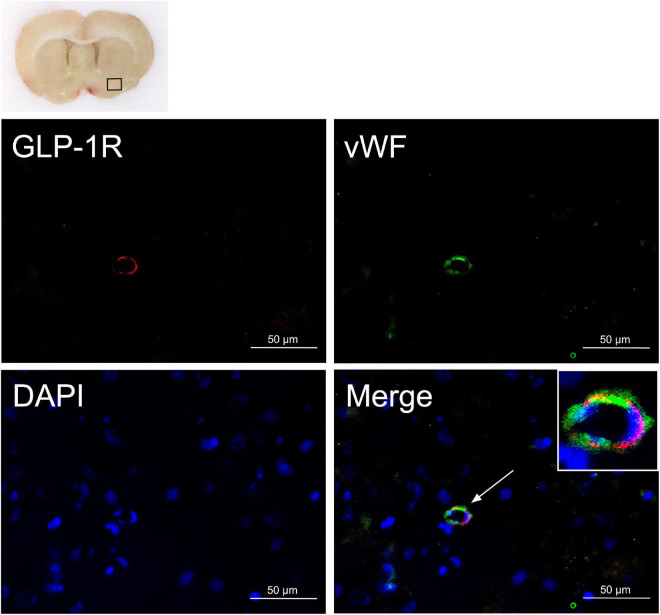
Endothelial expression of GLP-1R after SAH. Colocalization of GLP-1R with endothelium (vWF, green) in microvessels at 24 h after SAH. Nuclei are stained with DAPI (blue). The top panel indicates the location of staining in the brain (small black box). Scale bar = 50 μm, *n* = 3. vWF, von Willebrand factor.

Double immunofluorescence of GLP-1R and vWF was performed on the brain sample sections collected 24 h after SAH induction. Colocalization of GLP-1R with endothelial cells (vWF) was observed on microvessels (Figure 2) of the ipsilateral basal cortex.

### Exendin-4 Ameliorated Short-Term Neurobehavioral Deficiency After Subarachnoid Hemorrhage

**FIGURE 3:**
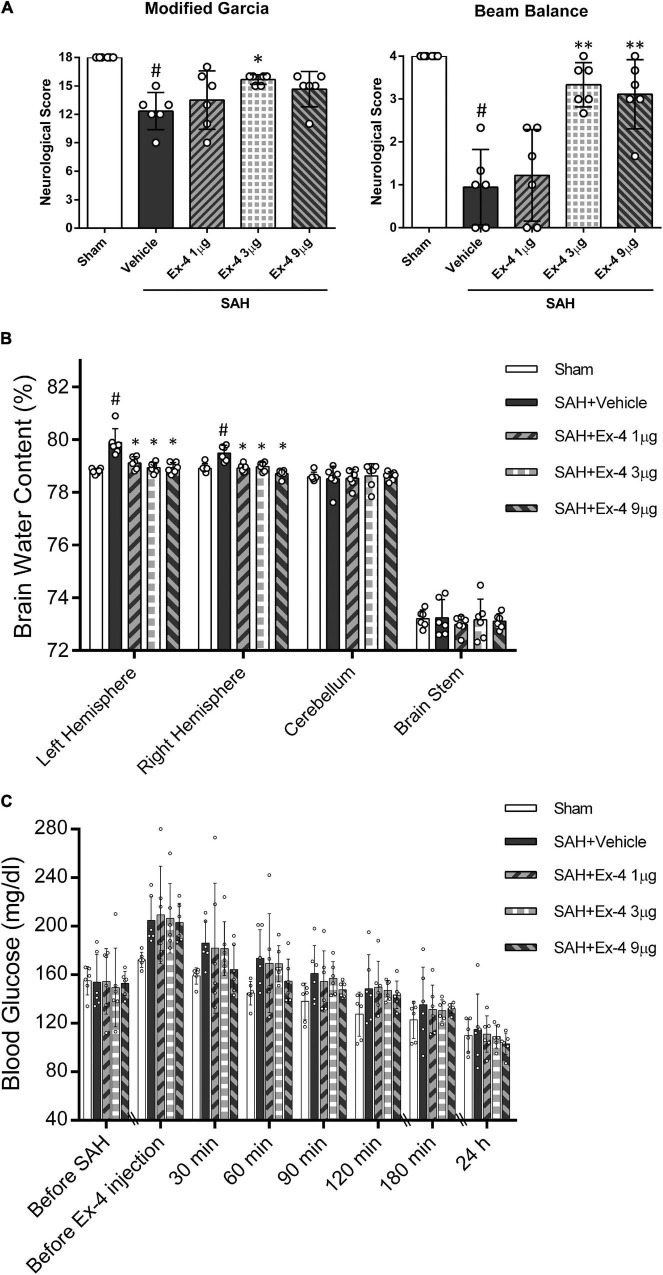
The effects of Exendin-4 (Ex-4) on neurobehavioral function, BWC, and blood glucose level after SAH **(A)** Exendin-4 improves the modified Garcia and beam balance score 24 h after SAH. **(B)** Administration of Ex-4 reduced BWC (%) in the left and right hemispheres at 24 h after SAH. **(C)** The effects of Ex-4 on blood glucose level after SAH. There is no significant difference in blood glucose change between SAH + Ex-4 (1, 3, and 9 μg/rat) group and the SAH + Vehicle group at each time point (before SAH, before Ex-4 injection, 30, 60, 90, 120, and 180 min and 24 h). *P < 0.05 vs. vehicle group; ^**^P < 0.01 vs. SAH + Vehicle group; ^#^*P* < 0.05 vs. sham group. Data were presented as mean ± SD. *n* = 6 per group.

Significantly reduced neurological scores of modified Garcia (*P* < 0.05, left panel) and beam balance (*P* < 0.05, right panel) were observed at 24 h after surgery in the SAH + Vehicle group compared with the sham group. Administration of medium-dose Ex-4 (3 μg) significantly improved the neurological scores in both tests compared with the SAH + Vehicle group after SAH (*P* < 0.05) (Figure 3A).

### Exendin-4 Attenuated Neuronal Brain Edema After Subarachnoid Hemorrhage

Subarachnoid hemorrhage induction significantly increased the brain edema in both left and right hemispheres in the SAH + Vehicle group compared with the sham group at 24 h after SAH (*P* < 0.05). The increase in brain edema in both hemispheres was considerably attenuated in all three SAH + Ex-4 groups (1, 3, and 9 μg) compared with the SAH + Vehicle group (*P* < 0.05). There was no significant difference in BWC in the cerebellum and brain stem across all groups (Figure 3B).

### The Effects of Exendin-4 on Blood Glucose Level

Plasma blood glucose levels of animals enrolled for BWC measurement were monitored from the start of surgery to 24 h after SAH induction. The serial change of blood glucose was demonstrated in Figure 3C. Overall, no statistical difference in blood glucose was observed between all SAH + Ex-4 (1, 3, and 9 μg) groups and SAH + Vehicle group at each measured time point (before SAH, before Ex-4 injection, 30, 60, 90, 120, and 180 min and 24 h) (*P* > 0.05). A trend was observed in the SAH + Ex-4 (9 μg) group with a rapid decrease compared with other SAH groups after drug treatment. However, these differences in blood glucose levels did not reach significance (*P* > 0.05) compared with the SAH + Vehicle group after SAH. The values of blood glucose were not lower in the SAH + Ex-4 (9 μg) group compared with those in the sham group at each time-point during the first 180 min after SAH (*P* > 0.05). Thus, to reduce the potential risk of hypoglycemia and maximize the neurological benefits, 3 μg of Ex-4 was determined as the best dose for the subsequent experiments.

### Exendin-4 Ameliorate Blood-Brain Barrier Permeability After Subarachnoid Hemorrhage

**FIGURE 4:**
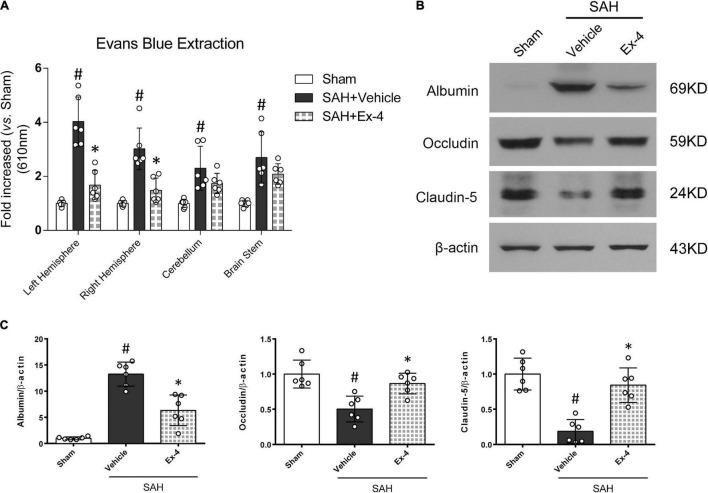
The effects of Ex-4 on blood-brain barrier (BBB) permeability at 24 h after SAH. **(A)** The effects of Ex-4 on Evans blue extravasation results at 24 h after SAH. **(B)** Representative Western blots band of BBB permeability-related proteins at 24 h after SAH. **(C)** Densitometric quantification of BBB permeability-related proteins. Ex-4 downregulated expression of albumin and upregulated expression of tight-junction-related proteins (Occludin and Claudin-5) in the SAH + Ex-4 group compared with the SAH + Vehicle group. β-actin was used as the loading control. *P < 0.05 vs. SAH + Vehicle group; ^#^*P* < 0.05 vs. sham group. Data were presented as mean ± SD. *n* = 6 per group. Ex-4, 3 μg/rat.

Extraction of Evans blue and Western blots on BBB-associated proteins were performed to evaluate the BBB permeability after SAH. The results from Evans blue extraction indicated a significant increase in the amount of Evans blue dye that had permeated to both hemispheres, cerebellum and brain stem, through the BBB (*P* < 0.05). However, the extravasation of Evans blue dye in both hemispheres was significantly reduced in the SAH + Ex-4 (3 μg) group compared with the SAH + Vehicle group (*P* < 0.05) (Figure 4A). Moreover, Western blots detected an increased expression of albumin and decreased expression of tight-junction-related proteins (Occludin and Claudin-5) in the SAH + Vehicle group (*P* < 0.05) compared with the sham group, while this effect was reversed in the SAH + Ex-4 (3 μg) group compared with the SAH + Vehicle group (*P* < 0.05) (Figures 4B,C).

### The Effects of Glucagon-Like Peptide 1 Receptor Small Interfering Ribonucleic Acid and Dorsomorphin on Neurological Function in Exendin-4-Treated Animals at 24 h After Subarachnoid Hemorrhage

**FIGURE 5:**
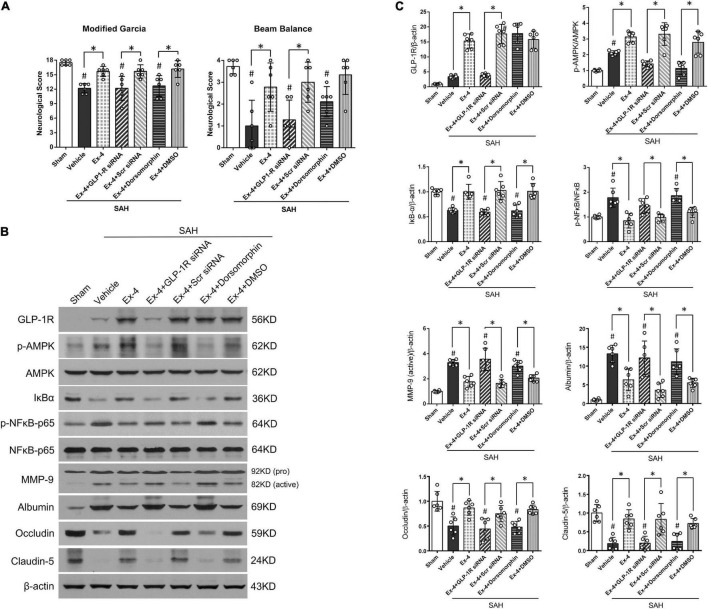
A possible molecular mechanism of Ex-4 on the BBB protection after SAH. **(A)** The effects of siRNA for GLP-1R, a GLP-1R siRNA, and Dorsomorphin, a specific inhibitor of AMPK, on modified Garcia score and beam balance score at 24 h after SAH. **(B)** Representative Western blots band of downstream signaling molecules of GLP-1R. **(C)** Densitometric quantification of downstream signaling molecules. *P < 0.05 between indicated groups; ^#^*P* < 0.05 vs. sham group. Ex-4, 3.0 μg/rat. DMSO, dimethyl sulfoxide; Scr siRNA, scrambled siRNA. β-actin was used as the loading control. Data were presented as mean ± SD. *n* = 6 per group.

Modified Garcia and beam balance tests were selected to evaluate the possible effects of GLP-1R siRNA and Dorsomorphin on neurological function after SAH. The modified Garcia score was significantly decreased in SAH + Ex-4 + GLP-1R siRNA and SAH + Ex-4 + Dorsomorphin groups when compared with SAH + Ex-4 + Scr siRNA and SAH + Ex-4 + DMSO groups, respectively (*P* < 0.05, left panel). In the beam balance test, administration of GLP-1R siRNA reversed the benefits of Ex-4 (3 μg) (*P* < 0.05, right panel), while administration of Dorsomorphin only partially reversed the benefits (*P* > 0.05, right panel) (Figure 5A).

### Exendin-4 Preserved Blood-Brain Barrier Integrity Through Glucagon-Like Peptide 1 Receptor/Activated Protein Kinase/Nuclear Factor-Kappa B Pathway

To investigate the potential mechanisms of Ex-4 in BBB protection, Western blot was performed on the downstream proteins of GLP-1R at 24 h after SAH (representative images in Figure 5B). Expression of GLP-1R slightly increased after SAH (*P* > 0.05) and further increased after administration of Ex-4 (3 μg) in SAH + Ex-4, SAH + Ex-4 + Scr siRNA, and SAH + Ex-4 + DMSO groups when compared with both the sham and SAH + Vehicle groups, respectively (*P* < 0.05) (Figure 5C). GLP-1R knockdown by GLP-1R siRNA efficiently inhibited the expression of GLP-1R in the SAH + Ex-4 + GLP-1R siRNA group compared with the SAH + Ex-4 + Scr siRNA group (*P* < 0.05). Consistent with the expression pattern of GLP-1R, expression of p-AMPK increased after SAH induction (*P* < 0.05) and further increased in the SAH + Ex-4 (3 μg) group compared with the SAH + Vehicle group. The phosphorylation of AMPK was significantly suppressed in SAH + Ex-4 + GLP-1R siRNA and SAH + Ex-4 + Dorsomorphin groups compared with SAH + Ex-4 + Scr siRNA and SAH + Ex-4 + DMSO groups, respectively (*P* < 0.05). To explore the changes of NF-κB corresponding to the interventions used, we assessed the expression of total IκB-α, phospho-NF-κB (p-NF-κB) p65, and total NF-κB p65 by Western blots. After SAH induction, the expression of IκB-α significantly decreased, while the expression of p-NF-κB p65 significantly increased (*P* < 0.05); these changes were reversed by the administration of Ex-4 (*P* < 0.05). When the animals were pretreated with GLP-1R siRNA or Dorsomorphin, Ex-4 was unable to elevate IκB-α expression or reduce p-NF-κB p65 expression as it was able to in SAH + EX-4 + Scr SiRNA and SAH + Ex-4 + DMSO groups. Paralleling the expression trend of p-NF-κB p65, active MMP-9 significantly increased after SAH but was attenuated in the SAH + Ex-4, SAH + Ex-4 + Scr siRNA, and SAH + Ex-4 + DMSO groups compared with the SAH + Vehicle, SAH + Ex-4 + GLP-1R siRNA, and SAH + Ex-4 + Dorsomorphin groups (*P* < 0.05). Next, BBB-associated proteins were measured. The reduced expression of albumin in the Ex-4 (3 μg) group was significantly upregulated in the SAH + Ex-4 + GLP-1R siRNA and SAH + Ex-4 + Dorsomorphin groups compared with the SAH + Ex-4 + Scr siRNA and SAH + Ex-4 + DMSO groups, respectively (*P* < 0.05). Expression of Claudin-5 and Occludin, tight-junction-related proteins, were downregulated by the pretreatment of GLP-1R siRNA and Dorsomorphin when compared with negative controls (*P* < 0.05) (Figures 5B,C).

**FIGURE 6:**
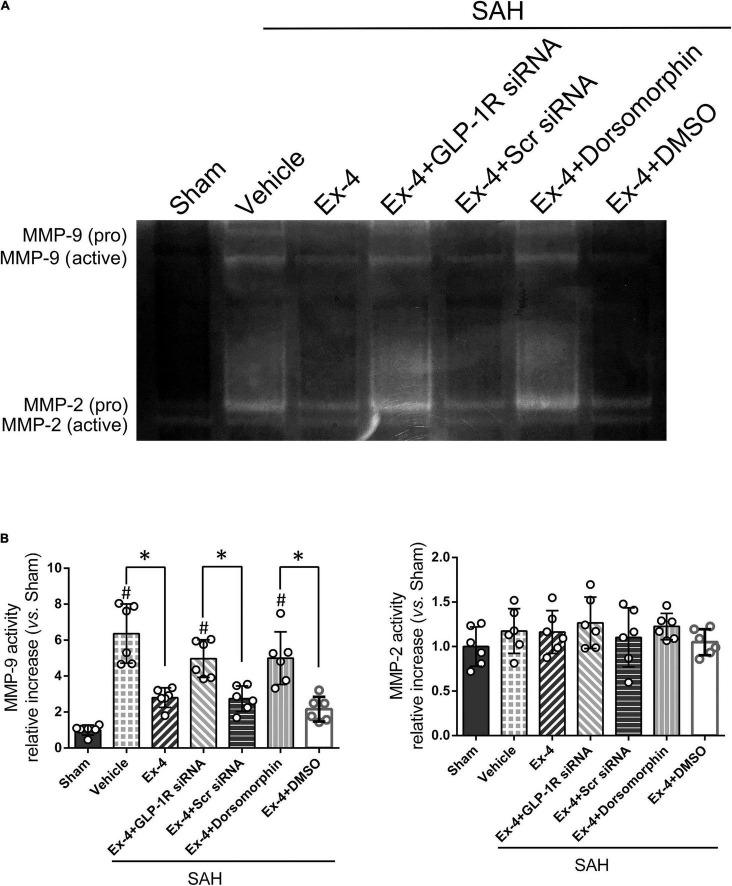
The effect of Ex-4 on the activity of the MMPs at 24 h after SAH. **(A)** Representative gelatin zymography demonstrates the activity of MMP-9 and MMP-2. **(B)** Quantification of active MMP-9 and MMP-2 on gelatin zymography. Ex-4, 3.0 μg/rat. MMPs, matrix metalloproteinases; Scr siRNA, scrambled siRNA; Dorsomorphin, a specific inhibitor of AMPK; DMSO, dimethyl sulfoxide. The activity of MMPs was represented as the intensity ratio of active form/proform. Data were presented as mean ± SD. *P < 0.05 between indicated groups; ^#^*P* < 0.05 vs. sham group. *n* = 6 per group.

Gelatin zymography was performed to confirm the activity of MMP-9 related to its expression in the Western blot results. MMP-9 activity, but not MMP-2, significantly increased at 24 h after SAH (*P* < 0.05). Elevated activity of MMP-9 after SAH was suppressed in the SAH + Ex-4 + Scr siRNA and SAH + Ex-4 + DMSO groups compared with the SAH + Ex-4 + GLP-1R siRNA and SAH + Ex-4 + Dorsomorphin groups, respectively (*P* < 0.05) (Figures 6A,B).

## Discussion

**FIGURE 7:**
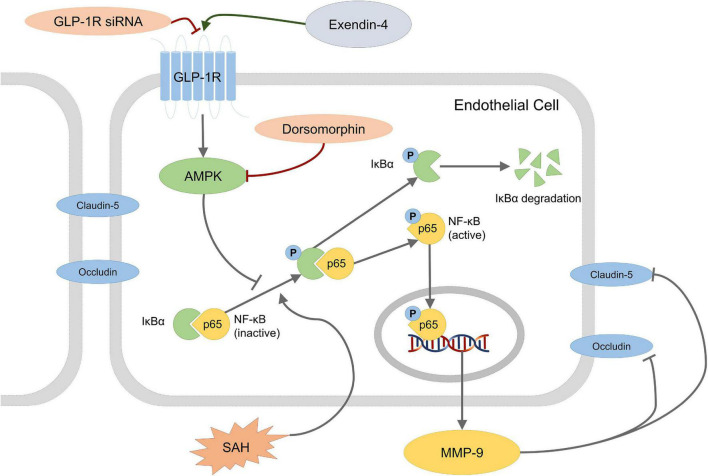
Schematic presentation of a possible mechanism of Ex-4 action on BBB disruption after SAH. Dorsomorphin, a specific inhibitor of AMPK; NF-κB, nuclear factor-kappa B; MMP-9, matrix metalloproteinases-9.

In the present study, we investigated the role of Ex-4 on the protection of BBB integrity after SAH. The major findings were listed as following: (1) GLP-1R is expressed on endothelial cells in microvessels of the brain after SAH; (2) administration of Ex-4 reduced brain edema in both hemispheres and improved neurological function after SAH without increasing risk of hypoglycemia; (3) administration of Ex-4 ameliorated BBB disruption after SAH; (4) the ability of GLP-1R siRNA and Dorsomorphin interventions to reverse the effects of Ex-4 suggests that the preserved BBB integrity might be due to the downstream GLP-1R/AMPK activation and inhibition of MMP-9 after SAH (Figure 7).

In the past few years, other studies of GLP-1R have suggested this receptor to be a promising therapeutic target in various CNS diseases (Teramoto et al., 2011; Hou et al., 2012; Rachmany et al., 2013; Sato et al., 2013; Kuroki et al., 2016; Zhu et al., 2016). Ex-4, a specific agonist and GLP-1 analog, has been reported to exert neuroprotective effects *via* the direct binding with GLP-1R (Mann et al., 2007). In agreement with these studies, the pretreatment of GLP-1R siRNA abolished the neuroprotective benefits of Ex-4, strengthening the predicted relationship between Ex-4 and GLP-1R in the SAH model.

Adenosine monophosphate-activated protein kinase is a sensor of cellular energy levels and responds to low ATP/AMP ratios (Hardie, 2004). Literature has reported increased expression of AMPK after SAH and may be responsible for the impaired energy metabolism in EBI (An et al., 2015). Specifically, mammal-originated AMPK consists of a catalytic α subunit and regulatory β and γ subunits (Hardie, 2003). Emerging evidence has indicated that GLP-1R can phosphorylate the AMPKα subunit in several cell types (Beiroa et al., 2014; Wang and Yang, 2015), including endothelial cells (Krasner et al., 2014; Koska et al., 2015; Wei et al., 2016). Endothelial AMPKα1 is one of the main regulators of endothelium-derived factors (Enkhjargal et al., 2014) and is important in cerebral vascular regulation (Enkhjargal et al., 2017). Though the detailed mechanism underlying BBB protection remains unclear, increased intracellular cAMP and Ca^2+^ levels, mediated by GLP-1R activation, may be involved in the activation of AMPK (Krasner et al., 2014; Liu et al., 2014).

It is reported that NF-κB p-65 is activated and transferred into the nucleus after SAH (Won et al., 2015; Wu et al., 2017). NF-κB in the nucleus increases the expression of MMP-9 by attaching to its binding site (Sato and Seiki, 1993; Fini et al., 1994). In this study, decreased expression of IκB-α and increased expression of p-NF-κB p65 and MMP-9 were found in the SAH + Vehicle group, consistent with previous results. Moreover, it was believed that the phosphorylated AMPK would inhibit the activation of NF-κB by interfering with the phosphorylation of IκB-α (Salminen et al., 2011), which was supported by our results, attenuated expression of p-NF-κB p65 and MMP-9 in the Ex-4 group compared with the control group. Correspondingly, results in the gelatin zymography indicated that the activity of MMP-9, along with its expression, was inhibited by the administration of Ex-4 possibly through an AMPK/NF-κB-dependent regulation.

Since pretreatment with GLP-1R siRNA and Dorsomorphin reversed the effects of Ex-4, and its expression of downstream proteins, we believe that the GLP-1R/AMPK/NF-κB signaling pathway is involved in neuroprotection after SAH. Previous studies reported that GLP-1R is expressed on neurons as well as astrocytes and microglia cells after inflammation (Alvarez et al., 1996; Gu et al., 2013). Additionally, MMP-9 is expressed in neurons, astrocytes, and microglia cells following SAH (Ying et al., 2016). Therefore, the beneficial effects of EX-4 *via* GLP-1R/AMPK-dependent NF-κB/MMp-9 inhibition pathway might also be involved in neurons, astrocytes, and microglia cells after SAH.

There are a few limitations to this study. First, although it has been reported that a single injection of Ex-4 can improve endothelial function (Koska et al., 2015) and protect BBB integrity in ischemic stroke (Kuroki et al., 2016), we did not try to explore the potential benefits of multiple injections of Ex-4. Second, this study did not assess the possible neuroprotective effects of Ex-4 in other pathophysiological processes in EBI, namely, apoptosis and inflammation. A previous study suggested that prolonged AMPK phosphorylation may promote neuronal apoptosis after SAH (An et al., 2015). Despite the possible proapoptotic effects of AMPK, the overall neurological function improvements indicated a neuronal protective role for Ex-4. To reveal the underlying mechanisms of Ex-4 in EBI after SAH, further studies are expected to explore the alternative pathways. Third, it has been reported that GLP-1R agonists could promote vasodilation in large vessels *via* the AMPK/eNOS pathway (Koska et al., 2015; Wei et al., 2016; Ke et al., 2017). However, in this proposal, we focused on microvessels and BBB integrity. Furthermore, reversal of the advantageous effects of EX-4 by GLP-1R siRNA and Dorsomorphin interventions suggests that the GLP-1R/AMPK/NF-κB pathway may be critical in preserving the BBB integrity after SAH. Fourth, this study did not perform an experimental control with AICAR, a non-specific agonist of AMPK, for its potential non-specific effects on proinflammatory cytokines such as TNF-α and mTOR signaling pathway in an AMPK independent manner (Rao et al., 2016). Fifth, we had aware that Dorsomorphin, as an AMPK inhibitor, actually might affect several relevant kinases, which may lead to non-specific inhibition. However, we would like to repeat the experiment when a more selective analog of Dorsomorphin is available online (Machrouhi et al., 2010). In this study, we believed that through a non-specified effect that might happen, Dorsomorphin did achieve AMPK inhibition with altered downstream protein expression after SAH. In that case, we proposed that GLP-1R activation regulates NF-K b/MMP-9 inhibition through AMPK. Finally, though lower glucose level was not detected in the SAH + Ex-4 (9 μg) group (Figure 3C), a higher dose of Ex-4 (>9 μg) might lead to hypoglycemia or energy metabolism alternation in endothelial cells, which could abolish the protective effect. Further study would set more gradient dose groups to explore this issue and evaluate the safety of Ex-4 administration after SAH.

In summary, this study suggested that Ex-4 could preserve the BBB integrity and improve neurological function after SAH. GLP-1R/AMPK-dependent NF-κB/MMP-9 inhibition might be involved as an important mechanism of neuroprotection by Ex-4, making GLP-1R a potential therapeutic target in EBI after SAH.

## Data Availability Statement

The original contributions presented in the study are included in the article/supplementary material, further inquiries can be directed to the corresponding authors.

## Ethics Statement

The animal study was reviewed and approved by Institutional Animal Care and Use Committee (IACUC) of Loma Linda University.

## Author Contributions

ZX, JT, and JZ made the idea and experimental design of this study. ZX, LW, QZ, and TZ performed most of the experiments. ZX and BE drafted the manuscript. ZX and JZ approved the final version of the manuscript on behalf of all the authors. All authors did the critical revision and language modification of the manuscript.

## Conflict of Interest

The authors declare that the research was conducted in the absence of any commercial or financial relationships that could be construed as a potential conflict of interest.

## Publisher’s Note

All claims expressed in this article are solely those of the authors and do not necessarily represent those of their affiliated organizations, or those of the publisher, the editors and the reviewers. Any product that may be evaluated in this article, or claim that may be made by its manufacturer, is not guaranteed or endorsed by the publisher.
